# Supplementary material for: Empirical methods for controlling false positives and estimating confidence in ChIP-Seq peaks
Source: BMC Bioinformatics. 2008 Dec 5;9:523. doi: 10.1186/1471-2105-9-523 (PMC2628906; doi:10.1186/1471-2105-9-523)
Supplement: Additional File 1 — A variety of html documents from the USeq web site detailing the available applications, their best usage, output file type descriptions, command line menus, etc. [file 1471-2105-9-523-S1.zip › USeqUserGuides/JavaDoc.zip!/JavaDoc/util/bio/parsers/package-use.html]

Uses of Package util.bio.parsers


|  |  |  |
| --- | --- | --- |
| **Overview** \| **Package** \| Class \| **Use** \| **Tree** \| **Deprecated** \| **Index** \| **Help** | |  |
| PREV   NEXT | **FRAMES**    **NO FRAMES**     **All Classes** |  |


---


## **Uses of Package util.bio.parsers**

| Packages that use util.bio.parsers | |
| --- | --- |
| **gata.aligner** | For aligning and post processing DNA sequences using BLAST for subsequent import into GATAPlotter. |
| **trans.main** | Principle classes and scripts used in TiMAT2 analysis. |
| **trans.misc** | Rough working scripts. |
| **trans.roc** | For manipulating the BAC dmel spike in data and generating ROC curves. |
| **util.bio.annotation** | For representing gene annotation. |
| **util.bio.parsers** | Parsers and extractors, fasta, quality, text, gff.... |

Classes in util.bio.parsers used by gata.aligner

****MultiFastaParser****
  
          Extracts sequences and headers out of multi fasta files.

Classes in util.bio.parsers used by trans.main

****UCSCGeneLine****

Classes in util.bio.parsers used by trans.misc

****UCSCGeneLine****

Classes in util.bio.parsers used by trans.roc

****UCSCGeneLine****

Classes in util.bio.parsers used by util.bio.annotation

****UCSCGeneLine****

Classes in util.bio.parsers used by util.bio.parsers

****Fasta****

****UCSCGeneLine****

---


|  |  |  |
| --- | --- | --- |
| **Overview** \| **Package** \| Class \| **Use** \| **Tree** \| **Deprecated** \| **Index** \| **Help** | |  |
| PREV   NEXT | **FRAMES**    **NO FRAMES**     **All Classes** |  |


---
